# The Impact of Physical Exercise on microRNAs in Hemodialysis Patients: A Review and a Protocol for an Ancillary Study

**DOI:** 10.3390/biomedicines12020468

**Published:** 2024-02-19

**Authors:** Rossella Elia, Giovanni Piva, Francesca Bulighin, Nicola Lamberti, Fabio Manfredini, Giovanni Gambaro, Alessio Di Maria, Gianluca Salvagno, Luca Giuseppe Dalle Carbonare, Alda Storari, Maria Teresa Valenti, Yuri Battaglia

**Affiliations:** 1 PhD Program in Clinical and Experimental Biomedical Sciences, Department of Medicine, University of Verona, 37129 Verona, Italy; rossella.elia@univr.it; 2 PhD Program in Environmental Sustainability and Wellbeing, Department of Humanities, University of Ferrara, 44122 Ferrara, Italy; giovanni.piva@unife.it; 3 Nephrology and Dialysis Unit, Pederzoli Hospital, 37019 Peschiera del Garda, Italy; f.bulighin@gmail.com; 4 Department of Medicine, University of Verona, 37129 Verona, Italy; 5 Department of Neuroscience and Rehabilitation, University of Ferrara, 44122 Ferrara, Italy; nicola.lamberti@unife.it (N.L.); fabio.manfredini@unife.it (F.M.); 6 Nephrology and Dialysis Unit, Department of Medicine, University of Verona, 37129 Verona, Italy; giovanni.gambaro@univr.it; 7 Nephrology and Dialysis Unit, University Hospital of Ferrara, 44122 Ferrara, Italy; alessio.dimaria@ospfe.it (A.D.M.); a.storari@ospfe.it (A.S.); 8 Section of Clinical Biochemistry, University of Verona, 37129 Verona, Italy; gianluca.salvagno@univr.it; 9 Internal Medicine, Section D, Department of Medicine, University of Verona, 37129 Verona, Italy; luca.dellecarbonare@univr.it (L.G.D.C.); mariateresa.valenti@univr.it (M.T.V.)

**Keywords:** physical activity, chondrogenesis, osteogenesis, vascular calcification, endothelial progenitor cells, endothelial dysfunction, exercise therapy

## Abstract

Physical inactivity is considered a significant risk factor for mortality and morbidity among chronic hemodialysis (HD) patients. Therefore, physical exercise is recommended in the treatment of HD patients. Although the beneficial effects of physical exercise in HD patients are well-described in the literature, the underlying physiological mechanisms still need to be fully understood. Recently, microRNAs (miRNAs) have emerged as potential mediators of the therapeutic effects of physical exercise in healthy individuals. miRNAs are short, single-stranded, noncoding RNAs involved in gene expression regulation. Specifically, upon forming the RNA-induced silencing complex, miRNAs selectively bind to specific miRNAs within cells, reducing gene expression. miRNAs can be secreted by cells in an accessible form or enclosed within exosomes or extracellular vesicles. They can be detected in various body fluids, including serum (circulating miRNAs), facilitating the study of their diverse expression. Currently, there is no available data regarding the impact of physical exercise on the expression of miRNAs involved in osteogenic differentiation, a fundamental mechanism in the development of vascular calcification, for HD patients. Therefore, we have designed an observational and longitudinal case-control study to evaluate the expression of miR-9 and miR-30b in HD patients participating in a 3-month interdialytic physical exercise program. This paper aims to present the study protocol and review the expression of circulating miRNAs in HD patients and their modulation through physical exercise.

## 1. Introduction

Extracorporeal hemodialysis (HD) is the most common kidney replacement therapy for patients with end-stage kidney disease (ESKD) [1,2]. Despite the use of efficacious drugs and highly efficient dialysis, patients undergoing maintenance HD have a high mortality rate compared to the general population [3].

Because sedentary behaviour is considered one of the factors contributing to mortality and unfavourable clinical outcomes in HD patients, physical exercise has been recommended [4,5] and successfully implemented in several clinical trials [6,7]. Over the past few years, encouraging clinical results [8] have been published regarding the improvements induced by physical exercise in chronic kidney disease (CKD) complications, including bone mineral disorders, inflammation, and sarcopenia [9]. However, the pathophysiological mechanisms underlying exercise and cellular targets still need to be completed [10].

Recently, a significant body of literature supports small noncoding RNAs as possible mediators of the therapeutic effects of physical exercise [11]. Their discovery has become feasible due to advancements in transcriptomic technology and high-throughput analysis. The main classes of small noncoding RNAs are microRNAs (miRNAs), small interfering RNAs (siRNAs), and piwi-interacting RNAs (piRNAs). Despite their distinct biogenesis, these three short pieces of single-stranded RNA have a similar action mechanism involving RNA–RNA base-pairing, which generally reduces post-transcriptional gene regulation [12,13].

Among the various noncoding RNAs, miRNAs (~22 nucleotide length) are particularly interesting due to their involvement in post-transcriptional regulation and their potential to be utilized as biomarkers. In particular, miRNAs have surfaced as novel regulators of biological processes across nearly all organ systems, and a growing body of research is establishing connections between disrupted miRNA function and various disease mechanisms. However, the utility of miRNAs as therapeutic biomarkers for assessing the impact of physical exercise on HD patients is still debated [14,15,16,17,18]. Therefore, this review discusses the modulation of circulating miRNAs in CKD patients on dialysis treatment who engage in physical exercise. Additionally, we describe our planned case-control study protocol, which will assess the expression of two circulating miRNAs in HD subjects undergoing interdialytic physical exercise training compared to controls.

## 2. Search Strategy and Selection Criteria

We searched the PubMed, Web of Science, Scopus, and Google Scholar databases for articles published from their inception to 31 July 2023. We used the following search terms: “chronic kidney disease”, “end-stage kidney disease”, “end-stage renal disease”, “kidney failure”, “renal replacement therapy”, “dialysis”, “hemodialysis”, “microRNA”, “exercise”, and “physical activity”. We primarily included articles published in the English language.

## 3. MicroRNA

**Figure 1:**
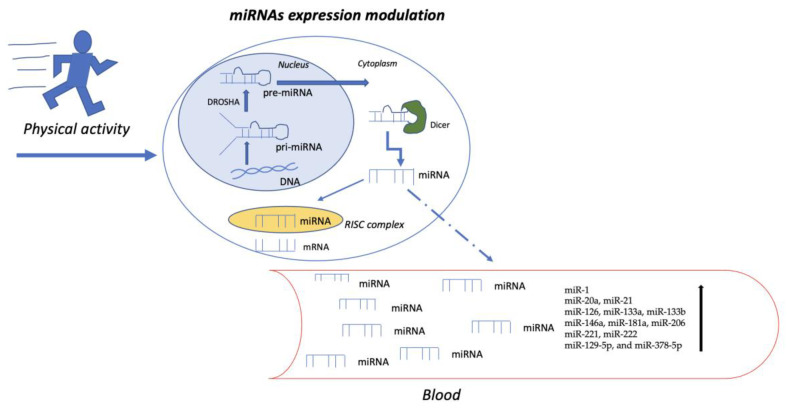
The figure illustrates the biogenesis of microRNAs, which begins with forming primary microRNA transcripts (pri-miRNA) and is subsequently processed by Drosha to generate pre-miRNA. Once exported to the cytoplasm, the Dicer complex is recruited to remove the stem-loop from pre-miRNA, forming mature miRNA. One strand of the miRNA duplex is then incorporated into the RNA-induced silencing complex (RISC). Within the RISC, miRNAs bind to complementary sequences on target mRNAs, leading to the repression of their translation or the induction of their degradation. However, cells can also secrete miRNAs and release them into circulation, contributing to cellular crosstalk and epithelial–mesenchymal transition, angiogenesis, fibrosis, inflammation, and osteogenic differentiation. Specifically, some of the miRNAs whose levels increase following physical activity are highlighted.

Over 1000 different miRNAs are synthesized from the human genome, and they can modulate one-third of human protein-coding genes [19]. The biogenesis of microRNAs involves an initial transcription by polymerase II (Pol II) to form primary-microRNA (pri-miRNA) transcripts, which are then processed by Drosha to generate pre-miRNAs. Exportin 5 (EXPO5) facilitates the export of pre-miRNAs from the nucleus to the cytoplasm. The Dicer complex is recruited to pre-miRNAs to excise the stem-loop, forming mature miRNAs, where one strand of the miRNA duplex is incorporated into the RNA-induced silencing complex (RISC) [20] (Figure 1).

Once formed, the RISC seeks out its target mRNAs by searching for complementary nucleotide sequences. The Argonaute protein, a component of RISC, holds the 5′ region of the miRNA to optimize its positioning for base-pairing with another RNA molecule. In animals, base-pairing typically involves at least seven nucleotide pairs and occurs most often in the 3′UTR of the target mRNA [21]. Once a miRNA binds an mRNA, several outcomes are possible. If the base-pairing is extensive, the Argonaute protein cleaves the mRNA, removing its poly-A tail and exposing it to exonucleases. After cleavage, the RISC complex and its associated miRNA are released to seek additional mRNAs [22].

The regulatory mechanism of miRNAs is similar to that of other RNAs, such as transcriptional activation or inhibition, epigenetic repression, and degradation. Intronic RNAs are often regulated by their host gene and processed from the intron, but they may also have an independent promoter region [22]. Multiple factors can account for the stability of microRNAs. The half-life of miRNAs can persist for five days or longer; however, some miRNAs have a rapid turnover [23].

The distinct miRNA expression profiles observed between normal and diseased tissues can serve as valuable diagnostic biomarkers [24]. MicroRNAs, whether released by cells in their free form or enclosed within vesicles, remain stable in bodily fluids, presenting a less invasive and more readily accessible alternative to biopsies [25]. MicroRNA has been isolated from saliva, blood (serum and plasma), faeces, urine, synovial fluid, follicular fluid, and pancreatic juice, and it is being examined for its utility as a biomarker for related diseases [26].

## 4. miRNAs in Chronic Kidney Disease

Some miRNAs have been recognized as potential biomarkers for enhancing diagnostic accuracy, predicting prognosis, and monitoring the course of kidney disease. In a recent study aiming to identify potential CKD biomarkers, miR-21, miR-17, and miR-150, three circulating miRNAs, were strongly associated with CKD in the Japanese population [27].

Furthermore, miRNAs are involved in various processes, including epithelial–mesenchymal transition (EMT), fibrosis, inflammation, and the activation of renal stem cells [28]. For instance, miR-155-5p has been implicated in promoting renal fibrosis under hypoxic conditions. It is transcriptionally regulated by p53 and regulates the cell cycle, cell growth, differentiation, and apoptosis. Upregulation of miR-155-5p may inhibit Sirt1, activate p53, and establish a positive feedback loop [29].

Studies have shown that exosomal miR-21 derived from tubular epithelial cells may accelerate the development of renal fibrosis by activating fibroblasts via the miR-21/PTEN/Akt pathway in obstructed kidneys [30]. Additionally, the expression of MiR-146a in the kidneys and its urinary excretion specifically correlates with the development of interstitial lesions and inflammatory cell infiltration [31].

The expression of other miRNAs, such as miR-16-5p, miR-17, miR-20a, and miR-106b-5p, decreases in small extracellular vesicles from CKD patients as kidney function deteriorates. Transfection of vascular smooth muscle cells (VSMCs) with each miRNA-mimic has been shown to mitigate calcification [32].

Recent studies have also indicated that specific miRNAs, namely miR-143, miR-145, and miR-223, can be increased in patients with CKD stages III-V and those treated with hemodialysis, while they decrease in renal transplant recipients [33]. Regarding miR-143 and miR-145, it is interesting to note that they play a role in vascular cell biology and are associated with CV disease [33]. So, it is not unexpected to find that they are dysregulated in a high-CV-risk condition, such as CKD. miR-223, a modulator of hematopoietic lineage differentiation involved in inflammatory and metabolic disorders, may contribute to the progression of chronic renal disease. Anglicheau et al. found elevated miRNA-223 levels in renal biopsies of patients with chronic progressive renal failure compared to patients with stable CKD [34].

## 5. miRNAs in CKD-Related Sarcopenia

Numerous investigations have focused on understanding the roles of miRNAs and long noncoding RNAs (lncRNAs) in skeletal muscle biology and the development of sarcopenia [35]. This degenerative process develops with age and is characterized by a loss of muscle mass and function.

Age-related sarcopenia is a consequence of altered target gene expression, which is influenced by the downregulation of various miRNAs and lncRNAs associated with muscle development and the upregulation of those linked to muscle atrophy. Specifically, activating the transforming growth factor-β (TGF-β) signalling pathway exacerbates sarcopenia while activating the insulin-like growth factor 1 (IGF-1) signalling system, the bone morphogenetic protein (BMP) signalling pathway, and the myogenic regulatory factor (MRF)-related signalling pathway alleviates it [36].

In patients with ESKD, especially those undergoing HD treatments, sarcopenia was found to increase the likelihood of adverse outcomes, including disability, metabolic dysfunction, reduced quality of life, and even mortality [9,37]. Changes in the expression levels of miRNAs and lncRNAs, which control several signalling pathways, contribute to muscle atrophy and sarcopenia [36,38] by either increasing protein degradation [39,40] or impairing myogenesis [41] in CKD settings.

MRFs, encompassing myogenic differentiation D (MyoD), myogenic factor 5 (Myf5), myogenin, and myogenic regulatory factor 4 (MRF4), play a crucial role in the process of skeletal myogenesis. When MyoD is not expressed, satellite cells exhibit enhanced expression of the paired box transcription factor (Pax7) while remaining dormant. MyoD and Myf5 facilitate the activation of satellite cells from their quiescent condition [42]. One particularly noteworthy subset of miRNA in skeletal muscle is myomiRs. Exosomal vesicles permit the production and transport of myomiRs. These miRNAs travel through the bloodstream as communicators and regulators in nearby muscle tissue and fat cells. myomiRs can regulate skeletal muscle plasticity by coordinating changes in muscle mass and fibre type in response to various contractile activities. Specifically, myomiRs are linked to the formation and maintenance of muscle mass in response to physical activity, the differentiation of satellite cells, the maintenance of physiological tropism, and the regulation of fibre switching. A group of miRNAs, including miR-23a, miR-26a, miR-29, miR-182, and miR-27a, acts as a potent regulator of muscle growth through the forkhead box protein O1 (FOXO1) pathway, phosphatase and tensin homolog (PTEN) genes and translational regulation, SMAD-2/3, and myostatin signalling [43,44,45]. These miRNAs are downregulated, enhancing protein degradation [39,40,41].

Additionally, malnutrition is associated with sarcopenia through the effect of miRNAs [46]. For instance, in plasma from sarcopenic patients with poor nutritional status, the downregulation of miR-206, which promotes myoblast differentiation by downregulating Pax7, has been observed. The regulation of miR-206, which depends on nutrient availability, may influence age-related muscle degeneration [47]. However, physical activity remains essential for reducing sarcopenia by regulating pro-inflammatory cytokines and miRNA levels through the IGF-1/AKT/mTOR signalling pathway [38,48].

Although sarcopenia is undoubtedly the result of several factors, its etiopathogenesis is still poorly understood. Therefore, identifying miRNAs might contribute to a better understanding of this phenomenon, even though the description of the myomiR profile is still in its early stages. This group of miRNAs appears to regulate satellite cell differentiation, overall proteostasis, muscle fibre structure and type, mitochondria and oxidative stress metabolism, the neurodegeneration process, and adipocyte infiltration into the skeletal muscle tissue of CKD patients.

## 6. miRNAs in Physical Activity and Exercise

Numerous studies have demonstrated that regular physical activity and exercise can significantly reduce the risk of cardiovascular disease, metabolic syndrome, and type 2 diabetes while also benefiting bone mineral density, muscle mass, and mood [49]. Even relatively short periods (less than four weeks) of bed rest without physical activity lead to negative structural and functional changes in various organs [50].

On the other hand, physical inactivity is typically exhibited in CKD patients, reaching its peak in HD patients. The low level of physical activity depends on many factors, including age, a high number of comorbidities, depression, fatigue, and sarcopenia. Furthermore, physical inactivity, together with obesity, smoking, and alcohol consumption, are major risk factors for chronic non-communicable diseases [51].

Patients with ESKD frequently have many comorbidities and risk factors. In the CHARES study, CV disease (24% and 12% in males and females, respectively), hypertension (78%, 71%), diabetes mellitus (33%, 24%), obesity (38%, 40%), and smoking (19%, 15%) were much more frequent than in the non-CKD population [52].

Thus, it is essential that stages of CKD patients, including HD patients, are engaged in appropriate levels of physical activity carried out with caution and in safe conditions [53].

### 6.1. Muscle Effect

Physical activity triggers the activation of second messengers, such as Ca++/CaMK, AMP/AMPK, and PKD. The activation of AMP/AMPK signalling through physical exercise determines the expression of glucose transporter type 4 (GLUT-4), peroxisome proliferator-activated receptor gamma coactivator 1-alpha (PGC-1α), and nuclear respiratory factor 1 (NRF-1). This effect, in turn, leads to the expression of genes involved in biogenesis and mitochondrial oxidative capacity [54]. Furthermore, physical activity induces epigenetic changes in the chromatin structure (methylation/histone acetylation), DNA methylation, and miRNA expression [55,56]. These mechanisms positively or negatively modulate the expression of the genes related to the proliferation of precursor cells, differentiation of microtubules, mitochondrial biogenesis and oxidative capacity, determination of muscle fibre type, mass maintenance and/or muscle hypertrophy, and muscle contractility [57].

On the other hand, physical exercise can modulate the gene expression profile in many cells and tissues [58], including the specific expression of miRNAs [59]. At the transcriptomic level, the activation of angiogenesis and tissue developmental networks during aerobic training predicts miRNAs’ modulation of RUNX2, SOX9, and PAX3 [57].

The regulation of gene expression is also influenced by the type of exercise, including endurance and resistance training [60]. In essence, endurance exercise, known as aerobic exercise, is defined by the American College of Sports Medicine as any activity that can be performed continuously using large muscle groups [61]. This exercise uses aerobic metabolism to engage and contract the muscle groups. On the other hand, resistance training encompasses exercises designed to increase the strength, power, hypertrophy, and/or endurance of specific muscles or muscle groups [62]. Indeed, this type of training stimulates the biosynthesis of contractile and structural proteins, leading to muscle hypertrophy and enhanced generation of contraction force. Furthermore, the satellite cells of skeletal muscles increase during resistance training due to the production of myokines, which are cytokine-like molecules secreted by skeletal muscles [63].

Aerobic physical exercise, for instance, increases the circulating plasma/serum levels of specific miRNAs (miR-1, miR-20a, miR-21, mir-126, miR-133a, miR-133b, miR-146a, miR-181a, miR-206, miR-221, and miR-222) in humans (Table 1 and Figure 1) [64,65].

Limited data on miRNA in HD patients who engage in exercise training are available. Resistance training during dialysis provides a non-pharmacological stimulus that may counteract decreases in protein synthesis and alterations in the activity of miRNAs caused by disease and treatment [28,29,30]. The effectiveness of exercise for improving health-related risks in both resistance and aerobic exercises during dialysis depends on the range of functional and metabolic adaptations of the muscle tissue [27].

Most HD patients have low exercise tolerance due to decreased muscle mass resulting from CKD-related catabolic status, cardiovascular complications, mitochondrial dysfunction, anaemia, and CKD-related mineral bone disorders [46]. Increasing or maintaining exercise tolerance is a critical factor in improving the quality of life of HD patients [66].

### 6.2. Biomineralization Phenomena

miRNAs play a role in regulating the osteogenic commitment of endothelial progenitor cells (EPCs) in response to physical exercise [67,68]. EPCs contribute to angiogenesis, vascular repair, and improved endothelial function, and reduced circulating EPCs are associated with vascular disease [69]. A recent study investigated the effect of physical exercise on the modulation of selected miRNAs in osteogenic differentiation using sera from half-marathon runners in cultured human mesenchymal stromal cells (MSCs). The results revealed increased expression of miR-21-5p, miR-129-5p, and miR-378-5p (Figure 1), which promote osteogenic differentiation, and reduced expression of miR-188-5p, involved in adipogenic progenitor cell differentiation. Additionally, downregulation of PTEN-SMAD7 expression and upregulation of protein levels of AKT/pAKT-SMAD4 along with RUNX2 was found in MSCs treated with post-run sera, highlighting the involvement of miR-21 in osteogenic differentiation [68].

Patients on dialysis treatment are susceptible to accelerated vascular calcification, defined as an inappropriate pathological deposition of calcium crystals in the vasculature, and, consequently, cardiovascular morbidity and mortality [70,71,72]. One mechanism is hyperphosphatemia-induced medial calcification, which involves the osteochondrogenic switch of VSMCs [73]. These actively participate in hydroxyapatite deposition in the extracellular matrix (ECM) caused by their osteoblastic-like trans-differentiation induced by high-Pi. This phenotypic switch can lead to changes in ECM characteristics that contribute to arterial stiffness [74]. Nonetheless, a distinct miRNA expression pattern emerges during osteogenic differentiation, indicating their essential role in bone formation [75]. Several miRNAs, including miR-128, mi-R130a-3p, miR-139-5p, and miR-378, are involved in this complex process by regulating the expression of various osteogenic proteins. Firstly, miR-128, if upregulated, increases the osteogenic differentiation of stem cells by enhancing the expression of alkaline phosphatase (ALP), the mineralization of the matrix, and the expression of the osteogenic proteins RUNX2, BMP-2, and COLA1 [76]. As a further effect, miR-128 enhances the activity of the Wnt/β-Catenin signalling pathway by targeting DKK2, an antagonist for this pathway [77]. Conversely, suppression of miR-128 inhibits the differentiation of osteoblasts. Secondly, miR-130a-3p can stimulate the osteogenic differentiation of ADSCs by reducing the expression of SIRT7, which subsequently enhances Wnt signalling-related proteins [78]. Thirdly, mirR-139-5p targets CTNNB1 and FZD4, which are essential molecules in the Wnt/β-Catenin cascade [79]. Lastly, miR-378 inactivates Wnt/β-Catenin signalling by suppressing the osteogenesis of human MSCs targeting Wnt6 and Wnt10a [57].

However, a growing interest in the scientific community has been directed towards two specific miRNAs, namely miR-9 and miR-30b. The first miRNA has been shown to modulate the proliferation of vascular smooth muscle cells in diabetic mice [80] and promote the osteoblast differentiation of mesenchymal stem cells in both human and murine models by binding to the 3’UTR region of DKK1 [81,82]. The second miRNA plays various biological roles, including the inhibition of proliferation, autophagy, apoptosis, and epithelial-to-mesenchymal and even osteoblastic transitions [83]. Notably, the downregulation of miRNA-30b has been demonstrated to induce the dedifferentiation of VSMCs into an osteoblastic-like phenotype by enhancing the expression of the Runx2 protein. Interestingly, Runx2 is a transcription factor expressed in response to pro-calcifying stimuli by osteoblastic-like VSMCs that activates the differentiation of osteoblasts and chondrocytes, a pivotal factor in promoting vascular calcification [84,85]. However, the effect of physical exercise on these miRNAs has yet to be investigated in CKD patients on dialysis.

## 7. Study Protocol

To the best of our knowledge, there is currently no available data regarding the effect of physical exercise on the expression of miRNAs involved in osteogenic differentiation in HD patients. We plan to conduct an observational and longitudinal case-control study to primarily evaluate the expression of circulating miRNAs, known as miR-9 and miR-30b, in patients on maintenance dialysis who practice physical exercise training compared to the control group.

This ancillary study of an ongoing trial [86] will enrol male subjects aged between 50 and 80 years with end-stage kidney disease undergoing hemodialytic treatment for at least three months who can walk for at least six meters. A Mini-Mental Status Examination score of ≥18 out of 30 will be required to ensure patients can give informed consent. Subjects with uncorrected anaemia (haemoglobin concentration <9 g/dL), acute infectious disease (C-reactive protein > 10 mg/L), uncontrolled hyperparathyroidism (both primitive and secondary), active oncologic disease, and severe cardio-respiratory concerns (e.g., unstable angina or severe heart failure identified by the New York Heart Association as class III-IV), as well as those with musculoskeletal or neurological conditions (e.g., lower limb major amputation) inhibiting exercise training, will be excluded. The Area-Vasta Emilia-Romagna Centro Ethics Committee (Bologna, Italy) approved the trial with the number 48/2019. A specific amendment was required and obtained for processing blood sample collection and analysis (EM169-2022_48/2019). We plan to enrol 15 patients (and 5 controls) in the pilot study.

### 7.1. Exercise Program

An exercise facilitator in the dialysis unit will administer a 3-month low-intensity exercise program. Each patient will be able to select the most appropriate training program that he/she prefers, choosing from a supervised or home-based program.

The supervised training will be carried out in the dialysis unit immediately before or after the dialysis session, and it will consist of 30-min sessions to be repeated 2 or 3 times per week.

The exercise regimen will include low-intensity walking, resistance and power exercises with elastic bands, ankle weights, and stretching for each session.

The training intensity will be set according to the patient’s baseline capacity and will be increased weekly.

Home-based training will consist of a semipersonalized walking program derived from previous experience with ESKD patients [87,88,89,90].

The program will involve a 10-min session daily, including intermittent walking with 1 or 2 min of work followed by a 1-min seated rest. The speed will be translated into a walking cadence, progressively increased weekly, and monitored at home using a metronome application on the patient’s smartphone.

More details related to the exercise programs are reported in the study protocol [91].

### 7.2. Control Group

A sample of HD patients with the same characteristics but who are unwilling to undertake any exercise intervention will be enrolled as a control group, and they will perform outcome measure sessions only, including blood sample collection.

### 7.3. Outcome Measures

The primary outcome will be miRNA analyses performed on samples collected during the short interdialytic period at baseline at the end of the program (3 months) and at a 6-month follow-up. As a secondary outcome, patients’ physical performance changes will be assessed through the 6-min walking test.

### 7.4. Blood Sample Collection and Analysis

Demographic data (comorbidities, dialysis vintage, cause of CKD), clinical parameters (systolic blood pressure, diastolic blood pressure, heart rate, Body Mass Index), and dialytic characteristics (urea reduction ratio and standard Kt/V urea calculation according to Daugirdas’ formula) will be collected at baseline. In addition, biochemical data, including haemoglobin, fasting glucose, creatinine, urea, sodium, potassium, calcium, phosphorus, uric acid, total proteins, albumin, vitamin D, and parathormone, will be tested.

To analyze miRNAs, peripheral blood samples (10 mL) will be collected and centrifuged at 400× *g*. miRNAs will be extracted from serum samples using the miRNeasy Serum/Plasma Advanced Kit (Quiagen Italia, Milano, Italy). The amount of RNA obtained will be quantified by measuring the absorbance at 260 nm. The purity of RNA will be checked by calculating the absorbance ratio at 260 nm compared to that at 280 nm, with a ratio ranging from 1.8 to 2.0 considered pure. miRNA analyses will be performed twice at baseline. A portion of the serum sample will be stored for future analysis.

First-strand cDNA will be synthesized according to the manufacturer’s protocol using the TaqMan microRNA Reverse Transcription Kit (Applied Biosystems Italia, Milano, Italy) with Reverse Buffer 10×, H2O, RNase Inhibitor, 4 dNTPs, specific primers, and RT Multriscribe.

The retrotranscription program will be 30′ 16 °C–30′ 42 °C–5′ 85 °C–4 °C (1.06 h). The resulting cDNA product will be aliquoted in equal volumes and stored at −20 °C. Real-time RT-PCR reactions will be carried out in multiplex. The real-time amplifications will include 10 min at 95 °C, 40 cycles at 95 °C for 15 s, and 60 °C for 1 min. The expression levels will be calculated for each sample in triplicate after normalization against the housekeeping genes (β_2_ microglobulin and GAPDH for mRNA or RNU44 for miRNAs) using the relative fold expression differences.

At baseline, a lateral plain X-ray of the lumbar spine and an echocardiogram will be performed to detect abdominal aortic and valvular calcification, respectively [91].

### 7.5. Statistical Analysis

The nominal variables will be presented as frequencies and percentages. The continuous variables will be reported as means with standard deviations or medians with interquartile ranges, depending on their distribution. Overtime variations in outcome measures, including miRNA expression, will be assessed through paired-sample tests according to data distribution. The comparison with the control group will be carried out via independent sample analyses. According to the study timeline, mixed models will be employed to assess the associations between the outcome measures and the clinical variables. A *p*-value of less than 0.05 will be considered statistically significant for all tests. The statistical analysis will be performed using SPSS 21.0.

## 8. Conclusions

Changing expressions of miRNAs can be used as valuable biomarkers for identifying skeletal muscle modulation during physical activity and exercise training in dialysis patients. Indeed, miRNAs can influence the gene expression involved in not only muscle mass, structure, and function but also in epithelial–mesenchymal transition, angiogenesis, fibrosis, inflammation, and osteogenic differentiation. These processes play a critical role in the development of vascular calcification.

In our study, we will address a gap in the current literature by assessing, for the first time, the expression of miR-9 and miR-30b among HD patients who will participate in a 3-month low-intensity exercise program. These two miRNAs transform mesenchymal cells into an osteogenic phenotype along blood vessels. The results of our study will furnish novel and innovative evidence concerning the potential utility of miRNAs as biomarkers for assessing cardiovascular risk in physical exercise among HD patients.

## Figures and Tables

**Table 1:** Levels of miRNAs modified in response to physical exercise.

miRNAs	Type of Exercise	Effects
miR-1	Acute endurance training Chronic endurance training	Increase
miR-16	Aerobic exercise	Increase
miR-7	Aerobic exercise	Decrease
miR-20a	Sustained rowing exercise training	Increase
miR-21	Acute endurance training Chronic endurance training	Increase
miR-29	Aerobic exercise	Decrease
miR-126	Acute endurance training Chronic endurance training	Increase
miR-133a	Acute resistance exercise Regular resistance exercise	Increase
miR-133b	Acute resistance exercise Regular resistance exercise	Increase
miR-146a	Acute exhaustive cycling exercise Sustained rowing exercise training	Increase
miR-148b	Aerobic exercise	Increase
miR-181a	Acute endurance training Chronic endurance training	Increase
miR-196b	Aerobic exercise	Increase
miR-206	Acute endurance training Chronic endurance training	Increase
miR-208-5p	Aerobic exercise	Increase
miR-221	Acute exhaustive cycling exercise	Increase
miR-222	Acute exhaustive cycling exercise Sustained rowing exercise training	Increase
miR-499	Aerobic exercise	Increase

## Data Availability

Not applicable.
